# Heat Shock Protein 27 Phosphorylation Regulates Tumor Cell Migration under Shear Stress

**DOI:** 10.3390/biom9020050

**Published:** 2019-01-30

**Authors:** Baohong Zhang, Fei Xie, Aziz ur Rehman Aziz, Shuai Shao, Wang Li, Sha Deng, Xiaoling Liao, Bo Liu

**Affiliations:** 1 School of Biomedical Engineering, Dalian University of Technology, Liaoning IC Technology Key Lab, Dalian 116024, China; zbhwwj@mail.dlut.edu.cn (B.Z.); xfscjc@mail.dlut.edu.cn (F.X.); azizjatoi@hotmail.com (A.u.R.A.); shuai.shao_ss@foxmail.com (S.S.); lw@mail.dlut.edu.cn (W.L.); dengsha@mail.dlut.edu.cn (S.D.); 2 Institute of Biomedical Engineering, Chongqing University of Science and Technology, Chongqing 401331, China; zxc_228@163.com

**Keywords:** heat shock protein 27, shear stress, migration, phosphorylation, signaling pathway

## Abstract

Heat shock protein 27 (HSP27) is a multifunctional protein that undergoes significant changes in its expression and phosphorylation in response to shear stress stimuli, suggesting that it may be involved in mechanotransduction. However, the mechanism of HSP27 affecting tumor cell migration under shear stress is still not clear. In this study, HSP27-enhanced cyan fluorescent protein (ECFP) and HSP27-Ypet plasmids are constructed to visualize the self-polymerization of HSP27 in living cells based on fluorescence resonance energy transfer technology. The results show that shear stress induces polar distribution of HSP27 to regulate the dynamic structure at the cell leading edge. Shear stress also promotes HSP27 depolymerization to small molecules and then regulates polar actin accumulation and focal adhesion kinase (FAK) polar activation, which further promotes tumor cell migration. This study suggests that HSP27 plays an important role in the regulation of shear stress-induced HeLa cell migration, and it also provides a theoretical basis for HSP27 as a potential drug target for metastasis.

## 1. Introduction

Metastasis, the most fatal characteristic of malignant tumors, accounts for more than 90% of tumor-related mortalities [1]. In hematogenous cancer metastasis, cancer cells first separate from the primary tumor and then degrade host stroma and intravasate into the blood and/or lymphatic vasculature. Finally these cells extravasate from endothelium tissues and invade parenchyma tissues of the distant organ [2]. Tumor cells must transit through microenvironments with dramatically varying physical forces to initiate the metastatic spread of cancer. For example, shear stress plays a vital role in cell behavior during metastasis [3]. It has been reported that hemodynamic shear stress stimulates migration of tumor cells as well as their extravasation by increasing cellular oxidative levels [4]. In addition, it also alters the interactions of tumor cells and improves their viability and proliferation [5,6]. Similarly, some reports have shown that low shear stress may endow tumor cell migration ability via activating the focal adhesion kinase (FAK)-extracellular regulated protein kinases 1/2 (ERK1/2) signaling pathway or Yes-associated protein 1 [7,8,9]. Furthermore, shear stress may enhance cell migration through the integrins-FAK-Rho GTPases signaling pathway in a time-dependent manner [7]. These discrepancies suggest that different shear stresses may affect migration ability through various pathways, while the specific underlying mechanism remains poorly understood.

Heat shock protein 27 (HSP27), also named HSPB1 by Kampinga et al. [10], belongs to a family of small heat shock proteins. It participates in the regulation of various physiological processes of cells under normal and stress conditions and is closely related to tumorigenesis and tumor development [11,12]. It is made up of a highly conserved α-crystallin domain, a less conserved N-terminal WDPF (W: tryptophan, D: aspartic acid, P: proline, and F: phenylalanine) domain, a partially conserved amino-terminal sequence PSRLFDQXFGEXLL, and a flexible carboxy-terminal region [13]. Phosphorylation of Ser15, Ser78, and Ser82 sites at the amino-terminal can regulate the degree of oligomerization of HSP27 and then up to 1000 kDa. This oligomerization is a highly dynamic process that seems to play a central role in regulating the chaperone activity of HSP27; the multimer has higher affinity for client proteins [14]. It has been reported that HSP27 is overexpressed in various cancer cells. Furthermore, it can protect tumor cells from apoptosis induced by various stimulations, reduce sensitivity of tumor cells to chemotherapy and radiotherapy, and regulate epithelial transformation [15]. Therefore, it has become a possible target for therapy [16].

The HSP27 is identified as a cap-protein of actin. It can stabilize the cytoskeleton, promote cell motility, and influence tumor cell invasion and metastasis [17]. Hepatocyte growth factor can induce HSP27 phosphorylation through the p38 mitogen-activated protein kinase (MAPK)-MK2 pathway, which in turn promotes metastasis [18]. Insulin-like growth factor 2 messenger RNA- (mRNA) binding protein 1 (IGF2BP1) facilitates the inhibition of MAPK4 mRNA translation, which interferes with MK5-directed phosphorylation of the HSP27. This limits G-actin sequestering by phosphorylated HSP27, enhances cell adhesion, and elevates the velocity of tumor cell migration [19]. The HSP27 expression and phosphorylation are related to cell migration and down-regulation of HSP27 expression may suppress cell migration. Moreover, growth factors can also induce cell migration through the activating p38MAPK-HSP27 pathway [20]. Similarly, shear stress can induce HSP27 phosphorylation [21]. To date, little is known about the effects of HSP27 on shear-stress-induced tumor progression and its mechanism. 

In this paper, a parallel plate flow chamber system is used to apply low shear force (5 dyn/cm^2^), normal shear force (20 dyn/cm^2^), and high shear force (40 dyn/cm^2^) to HeLa cells [3]. This system is suitable for the dynamic detection of living cells by microscopy through the fluorescence protein labeling technique and fluorescence resonance energy transfer (FRET) technique. Furthermore, this project is designed to observe the distribution and polymerization of HSP27 under shear stress, to detect its influence on downstream signal molecules, and finally to explore the molecular mechanism underlying shear stress for regulating cancer cell activity. It will be a step forward to understand the role of HSP27 on shear-stress-induced tumor progression and will be helpful for clinical trials.

## 2. Materials and Methods 

### 2.1. Establishment of Fluorescence Protein Fused Heat Shock Protein 27

Hochberg et al. have proved disulphide bond formation in the core domain of HSP27 [22] and self-association of wild type HSP27 has been confirmed by both sedimentation velocity and sedimentation equilibrium analysis [23]. Moreover, it has also been established that HSP27 forms an equilibrium mixture of monomers, dimers, tetramers, 12-mers, and 16-mers [23]. In addition, Lambert et al. have proposed that HSP27 forms stable dimers through the α-crystallin domain that further multimerize through intermolecular interactions mediated by the phosphorylation of a sensitive N-terminal domain [24]. Similarly, cell-free HSP27 monomers are also spontaneously changed to disulphide bond-linked dimers [25]. On the basis of the above data, we designed an intermolecular FRET biosensor in which one molecule is fused to a donor fluorescence protein (HSP27-ECFP) and another to an acceptor fluorescence protein (HSP27-ypet). The two molecules interact with each other and bring the donor and acceptor fluorescence proteins together for FRET generation. The FRET biosensor is highly dependent upon distance; if the distance is higher, FRET is not possible [26]. For this, fluorescence proteins (ECFP and Ypet) were fused to HSP27 and HSP27-ECFP and HSP27-Ypet plasmids were constructed through subcloning. The principle of this is shown in Figure S1: when two HSP27 molecules (HSP27-ECFP and HSP27-Ypet) interact with each other, the two fluorescent proteins ECFP and Ypet undergo the FRET phenomenon, and the ratio of Ypet/ECFP increases and confirms the self-polymerization within the whole domain. To examine the effect of phosphorylation on oligomerization, Ser15, Ser78, and Ser82 sites of HSP27-Ypet are mutated to Ala (HSP27-3A-Ypet) and similarly, HSP27-3A-ECFP is constructed too. Furthermore, Cyto-FAK and mCherry-actin plasmids are also used to explore focal adhesion kinase (FAK) activation and actin activation respectively [27,28]. The number of repeated experiments (*n*) is greater than five.

### 2.2. Cell Culture and Transfection

Before transfection, HeLa cells (Cell Resource Center of Shanghai Academy of Sciences, Chinese Academy of Sciences) were cultured with the high glucose version of Dulbecco’s modified Eagle medium (DMEM, Hyclone, Logan, UT, USA) containing 10% fetal bovine serum (FBS), 2 mmol/L L-glutamine, 100 unit/mL penicillin, and 100 mg/mL sodium pyruvate (GIBCO BRL, Grand Island, NY, USA). Lipofectamin 3000 (Invitrogen, Carlsbad, CA, USA) was chosen as a transfection reagent to transfect different DNA plasmids into HeLa cells. HeLa cells were passed onto fibronectin (abcam)-coated cover slips after transfection for 24 h and cultured with 0.5% FBS for 12 h before laminar flow application [29].

### 2.3. Flow Systems

Laminar flows were provided by a classic parallel-plate flow chamber which was modified to be suitable for dynamic observations under a FRET microscope [30]. Separated HeLa cells were seeded on a glass slide which was covered by a silicone gasket and a cover glass. Laminar shear stress was set to 5, 20, and 40 dyn/cm^2^ by adjusting fluid flow in the chamber [31]. The flow experiments were done at 37 °C with 5% CO_2_ to maintain the pH at 7.4 [29].

### 2.4. Transwell Migration Assay

In this study, the Transwell System (8-μm pore size polycarbonate membrane, Corning Inc., Corning, NY, USA) was used to evaluate HeLa cell migration. HeLa cells treated with inhibitors were added to the upper compartment of the chamber (in a 200 μL serum-free medium containing 2 × 10^4^ cells) and 600 μL 20% FBS medium was added to the lower chamber. After 24 h of incubation, cells which migrated to the bottom surface through the filter were fixed with 75% methanol for 15 min and then stained with Giemsa stain.

### 2.5. Image Acquisition

Image acquisition system included an inverted microscope (Nikon Eclipse Ti Series, Ti-Fl Epi-fl/1, Nikon, Tokyo, Japan) and a cooled charge-coupled device (Evolve-512-M-FW-16-AC, Photometrics, Tucson, AZ, USA). Time lapse fluorescence images were acquired with 60 s as the time interval via MetaMorph software (Universal Imaging, Downingtown, PA; USA), with a 440DF20 excitation filter for ECFP. A 455DRLP dichroic mirror and two emission filters were controlled by a filter controller (480DF30 for ECFP and 535DF25 for Ypet). Five time-lapse images were taken as the baseline before applying stimulation to the cells.

### 2.6. Image Analysis

Fluorescence resonance energy transfer images were processed using MetaMorph and MetaFluor (Universal Imaging) software and the spatiotemporal changes of fluorescent proteins in the cells were analyzed using Matlab (Mathworks, Natick, MA, USA). Firstly, all images were read and fluorescence intensity from the four corners of the image were calculated as the background. Then, background, noise, and edge recognition were removed. Images were divided into 50 parts equally along the direction of shear stress as explained in Supplementary Figure S2. In combination to time sequence of photographing, the fluorescence intensity of each part at the previous moment of stimulation was used as a reference to normalize fluorescence intensity, after which a three-dimensional graph of time, space, and fluorescence intensity was drawn and a color bar given at the right side of the images that mentions the range of the FRET ratio [32,33].

### 2.7. Statistical Analysis

Average fluorescence intensities of the 1st to 15th parts were taken as the parameters of the downstream distribution of the target protein and from the 36th to 50th parts average fluorescence intensities were used as the parameters of its upstream distribution. These two parameters were compared in Excel with *t*-tests to analyze the polarity of the target protein in the cell after 30 min of shear stress application. Significant differences were considered by *p* value (<0.05).

## 3. Results

### 3.1. Shear Stress Induces Heat Shock Protein 27 Polarization Distribution 

**Figure 1:**
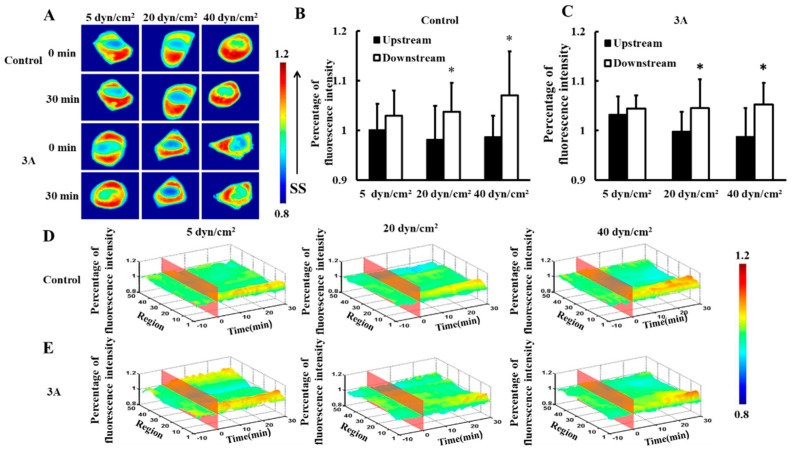
Effects of heat shock protein 27 (HSP27) phosphorylation on shear-stress-induced HSP27 polar distribution. (**A**) Fluorescence images of HeLa cells transfected with HSP27-Ypet (control) and HSP27-3A-Ypet (3A) plasmids before and after 30 min of 5, 20, and 40 dyn/cm^2^ shear stress stimulations. (**B**,**C**) The percentage of HSP27-Ypet and HSP27-3A-Ypet fluorescence intensity comparison of upstream to downstream in the control and 3A groups, respectively (* *p* < 0.05 when comparing to the upstream, with values taken from the 30 min time point). (**D**,**E**) The 3D distribution map of HSP27-Ypet and HSP27-3A-Ypet under 5, 20, and 40 dyn/cm^2^ shear stresses in the control and 3A groups, respectively. The number of repeated experiments (*n*) for 5, 20, and 40 dyn/cm^2^ in the control group was 14, 24, and 9, respectively, and *n* for the 3A group was 8, 9, and 11 for 5, 20, and 40 dyn/cm^2^, respectively).

To investigate the distribution of HSP27 in living cells under different magnitudes of shear stress, 5, 20, or 40 dyn/cm^2^ shear stresses were applied to HeLa cells transfected with HSP27-Ypet plasmid (control). There was no significance difference between upstream and downstream fluorescence under 5 dyn/cm^2^ shear stress within 30 min (Figure 1A,B, *p* > 0.05). However, HSP27 clustered at the downstream upon 20 and 40 dyn/cm^2^ shear stress applications within 30 min (Figure 1A). Thus, the fluorescence intensity of HSP27 was higher in the downstream than upstream (Figure 1B,D, *p* < 0.05). This shows that HSP27 is uniformly distributed in cells with low shear stress stimulation, while high shear stress distributes it polarly. Hence, shear-stress-induced polarity distribution of HSP27 is regulated by shear stress amplitude. 

To explore the effects of phosphorylation of HSP27 on shear-stress-induced HSP27 polarity distribution, HSP27-3A-Ypet (3A, non-phosphorylated variant) plasmids were transfected into HeLa cells. HSP27-3A-Ypet distribution showed similar distribution to the control group upon different shear stress applications (Figure 1A,C,E). Thus, HSP27 distribution under different mechanical conditions has no connection with its phosphorylation.

### 3.2. Shear-Stress-Induced Heat Shock Protein 27 Depolymerization is Regulated by its Phosphorylation

**Figure 2:**
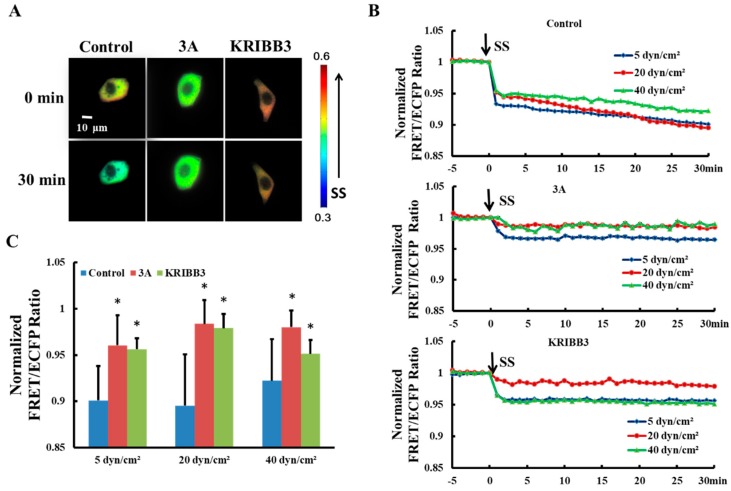
Effect of HSP27 phosphorylation on shear-stress-induced HSP27 depolymerization. (**A**) FRET ratio images of the control, 3A, and KRIBB3 groups before and after 20 dyn/cm^2^ of shear stress stimulation. (**B**) The fluorescence resonance energy transfer (FRET) ratio in the control, 3A, and KRIBB3 groups under different magnitude of shear stress (SS, from 0 min) after normalization. (**C**) The FRET ratio comparison in the control, 3A, and KRIBB3 groups (* *p* < 0.05 when comparing to the control group, with values taken from the 30 min time point). *n* for 5, 20, and 40 dyn/cm^2^ in the control group is 24, 12, and 15; for the 3A group it is 19, 15 and 13; and in the KRIBB3 group it is 17, 20, and 21, respectively.

It has been reported previously that intracellular HSP27 typically exists as a large oligomer and depolymerizes into smaller active molecules which are involved in the regulation of cell activity [34,35]. To explore the effects of shear stress on self-polymerization of HSP27 in living cells, HSP27-Ypet and HSP27-ECFP were co-transfected into Hela cells (control). The FRET ratios decrease by 10% (5 dyn/cm^2^), 11% (20 dyn/cm^2^), and 8% (40 dyn/cm^2^) within 30 min, respectively (Figure 2B), indicating that each shear stress can induce HSP27 depolymerization.

The function and conformation of HSP27 are regulated by its own phosphorylation [36]. To investigate the effect of HSP27 phosphorylation on its polymerization with shear stress stimulation, 5, 20, and 40 dyn/cm^2^ shear stresses were applied to HeLa cells co-transfected with HSP27-3A-Ypet and HSP27-3A-ECFP. The FRET ratio in HSP27 variants decreased less when compared to the control and the wild type HSP27 under 5, 20 and 40 dyn/cm^2^ of shear stress stimulations within 30 min (Figure 2B,C, *p* < 0.5). In addition, HeLa cells co-transfected with HSP27-Ypet and HSP27-ECFP were pretreated with KRIBB3 (inhibitor of HSP27 phosphorylation, 1 μM) [37,38] for 4 h before shear stress stimulation, with the down-regulation of HSP27 FRET ratio also rescued (Figure 2B,C, *p* < 0.05). Taken together, shear stress regulates the depolymerization of HSP27 through its own phosphorylation.

### 3.3. The Polarity Distribution of Actin in Response to Shear Stress is Regulated by Heat Shock Protein 27 Phosphorylation

**Figure 3:**
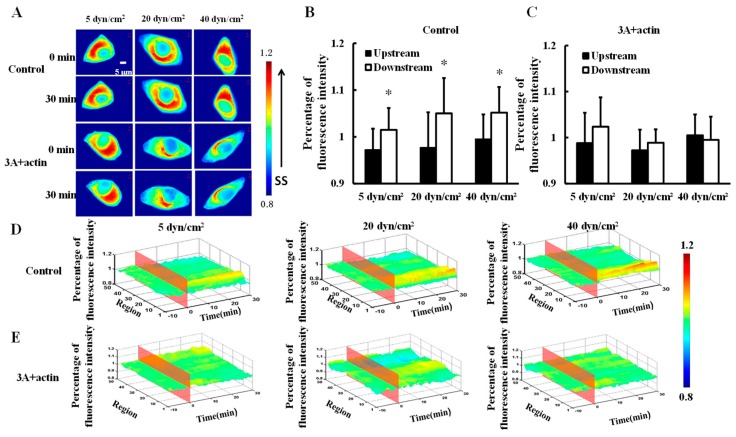
Effect of HSP27 phosphorylation on shear-stress-induced actin polar distribution. (**A**) Fluorescence images of HeLa cells transfected with mCherry-actin (control) and cells co-transfected with mCherry-actin and HSP27-3A (3A+actin) before and after 30 min of 5, 20, and 40 dyn/cm^2^ of shear stress stimulations. (**B**,**C**) The percentage of mCherry-actin fluorescence intensity comparison of upstream to downstream in the control and 3A+actin groups (* *p* < 0.05 when comparing to the upstream; values are from the 30 min time point). (**D**,**E**) The three dimensional (3D) distribution map of mCherry-actin under 5, 20, and 40 dyn/cm^2^ shear stresses in the control and 3A+actin groups (*n* for 5, 20, and 40 dyn/cm^2^ in the control group is 8, 14, and 11, and *n* in the 3A+actin group is 13, 9, and 12, respectively).

To investigate the effect of shear stress on actin distribution, mCherry-actin was transfected into HeLa cells and then 5, 20, or 40 dyn/cm^2^ shear stresses were applied to the transfected cells (control). Actin gradually clustered downstream (Figure 3A) and there were significance differences in fluorescence intensity between the upstream and downstream upon 30 min of 5, 2,0 or 40 dyn/cm^2^ shear stress applications (Figure 3B,D, *p* < 0.05). Moreover, HeLa cells were co-transfected with HSP27-3A and mCherry-actin (3A+actin) to investigate the effect of HSP27 phosphorylation on shear-stress-induced actin polar distribution. No significant differences in fluorescence intensity could be found between the upstream and downstream in the 3A+actin group (Figure 3A,C,E, *p* > 0.05). The results show that shear stress can induce polar distribution of actin, while it is uniformly distributed in the 3A+actin group. Finally, it may be concluded that shear-stress-induced actin polar distribution is regulated by HSP27 phosphorylation.

### 3.4. Expression and Phosphorylation of Heat Shock Protein 27 Regulates Shear-Stress-Induced Focal Adhesion Kinase Activation 

**Figure 4:**
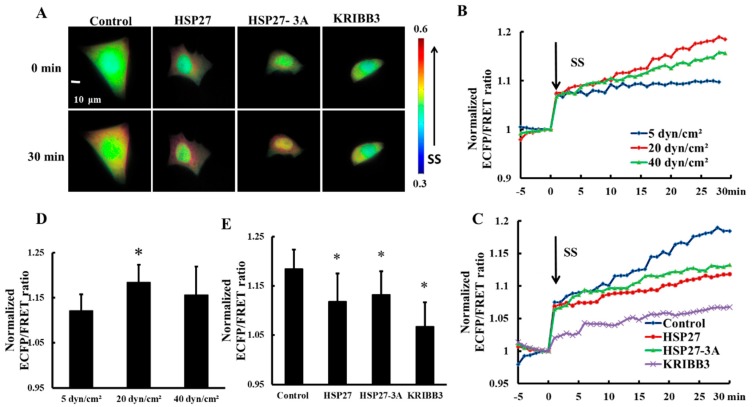
Effects of HSP27 phosphorylation on shear-stress-induced focal adhesion kinase (FAK) activation. (**A**) Living cell images of Cyto-FAK (control), Cyto-FAK co-transfected with HSP27 (HSP27), Cyto-FAK co-transfected with HSP27-3A (HSP27-3A), and Cyto-FAK transfected cells pretreated with 1 μM KRIBB3 (KRIBB3) under 20 dyn/cm^2^ of shear stress. (**B**) Cyto-FAK fluorescence resonance energy transfer (FRET) ratio under 5, 20, and 40 dyn/cm^2^ of shear stress (SS, from 0 min) stimulations after normalization. (**C**) The FRET ratio of Cyto-FAK in the control, HSP27, HSP27-3A, and KRIBB3 groups under 20 dyn/cm^2^ shear stress (SS, 0 min) stimulations after normalization. (**D**) The ratio comparison of Cyto-FAK in 5 (*n* = 9), 20 (*n* = 10) and 40 (*n* = 15) dyn/cm^2^ groups (* *p* < 0.05 when comparing to the 5 dyn/cm^2^ group). (**E**) The FRET ratio comparison in the control, HSP27, HSP27-3A, and KRIBB3 groups upon 30 min of 20 dyn/cm^2^ shear stress application (* *p* < 0.05 when comparing to the control and *n* in the HSP27, HSP27-3A, and KRIBB3 groups is 22, 7, and 14, respectively; values are taken from the 30 min time point).

Focal adhesion Kinase (FAK) is an important molecule through which tumor cells respond to extracellular mechanical stimulations and regulate cell behavior [39,40]. In order to explore the effect of shear stress on FAK activation, 5, 20, and 40 dyn/cm^2^ shear stresses were applied to HeLa cells transfected with Cyto-FAK plasmid. The FRET ratio of Cyto-FAK increased upon each shear stress application within 30 min (Figure 4B) and the FRET ratio increase under 20 dyn/cm^2^ of shear stress was the highest (Figure 4B,D, *p* < 0.05). The increase in FRET ratio of Cyto-FAK with 20 dyn/cm^2^ shear stress was then taken as the control. To explore the effect of HSP27 expression and phosphorylation on shear-stress-induced FAK activation, HSP27 and the non-phosphorylated variant HSP27-3A were co-transfected with Cyto-FAK into HeLa cells. The increase of FRET ratio in HSP27 overexpressed cells (the HSP27 group) with 20 dyn/cm^2^ shear stress was less than that in the control group (Figure 4A,C,E, *p* < 0.05). HeLa cells transfected with Cyto-FAK were pretreated with KRIBB3 (1 μM, 4 h) to inhibit HSP27 phosphorylation before shear stress application. The increase of FRET ratio in the KRIBB3 group was 12% lower than in the control group (Figure 4E, *p* < 0.05), which is similar to the HSP27-3A group. The results indicate that overexpression of HSP27 and inhibition of HSP27 phosphorylation can attenuate shear-stress-induced FAK activation.

**Figure 5:**
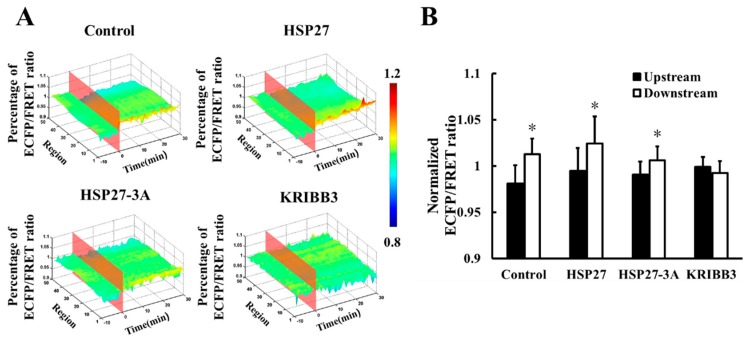
Effect of HSP27 on shear-stress-induced Cyto-FAK polar activation. (**A**) The 3D distribution of FRET ratio for the control, HSP27, HSP27-3A, and KRIBB3 groups with 20 dyn/cm^2^ shear stress stimulation. (**B**) Comparison of Cyto-FAK FRET ratio between upstream and downstream in the control, HSP27, HSP27-3A, and KRIBB3 groups upon 30 min of 20 dyn/cm^2^ shear stress application (* *p* < 0.05 when comparing to the upstream; values are from the 30 min time point).

To investigate the intracellular distribution of shear stress-induced FAK activation, the downstream and upstream FRET ratio of were analyzed using Matlab. It was found that the downstream FRET ratio of Cyto-FAK (control) was higher than that for the upstream after 20 dyn/cm^2^ shear stress for 30 min (Figure 5A,B, *p* < 0.05). A higher FRET ratio in the downstream of the HSP27 overexpressed group was also observed (Figure 5A,B, *p* < 0.05). In the co-transfected non-phosphorylated variant of HSP27, the FRET ratio in the downstream of Cyto-FAK was also found to be higher than the upstream, while its amplitude was significantly reduced (*p* < 0.05). However, the shear-stress-induced polar activation of Cyto-FAK disappeared when KRIBB3 was used to inhibit the phosphorylation of endogenous HSP27 (Figure 5A,B, *p* > 0.05). These results suggest that the polar activation of Cyto-FAK is regulated by HSP27 phosphorylation but it is not related to the expression level of HSP27.

### 3.5. Shear-Stress-Induced Heat Shock Proteain 27 Depolymerization is Regulated by Focal Adhesion Kinase Activation and Actin 

**Figure 6:**
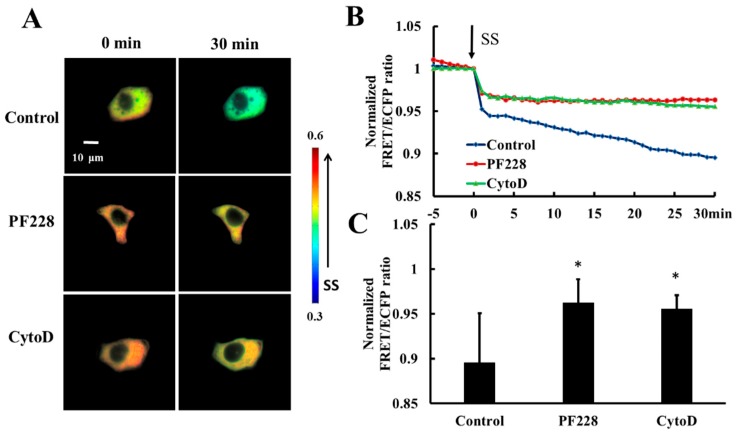
Effect of FAK activation and actin accumulation on shear-stress-induced HSP27 depolymerization. (**A**) The FRET ratio images of HSP27 under 20 dyn/cm^2^ of shear stress after pretreatment of 50 nM PF228 or 1 μM CytoD. (**B**) The line graph of HSP27 FRET ratio in the control, PF228, and CytoD groups upon 20 dyn/cm^2^ shear stress (SS, from 0 min). (**C**) HSP27 FRET ratio comparison in the control, PF228, and CytoD groups (* *p* < 0.05 when comparing to the control; values are from the 30 min time point). *n* is 12, 31, and 35 in the PF228 and CytoD groups, respectively.

HeLa cells co-transfected with HSP27-Ypet and HSP27-ECFP were pretreated with PF228 (FAK activation inhibitor, 50 nM) for 30 min [41,42] or Cytochalasin D (actin polymerization inhibitor, CytoD, 1µM) for 30 min [43] to explore the effect of FAK activation and actin polymerization on shear-stress-induced HSP27 depolymerization. The decrease in FRET ratio of HSP27-ECFP and HSP27-Ypet without any prior treatment was taken as the control. The FRET ratio in the control group decreased by 11% upon 20 dyn/cm^2^ shear-stress-stimulation within 30 min but decreased by 5% in the PF228 and CytoD groups (Figure 6A–C, *p* < 0.05). There was a significance difference in the FRET ratio between cells treated with inhibitor and the control (Figure 6C, *p* < 0.05). Taken together, the phosphorylation of HSP27 under shear stress stimulation does not unidirectionally regulate the activation of FAK and actin polymerization. Further, the cytoskeleton reorganization and FAK activation can also participate in the phosphorylation and depolymerization of HSP27 conversely.

### 3.6. Heat Shock Protein 27 Phosphorylation is Closely Related to Cell Migration with Shear Stress Stimulation

**Figure 7:**
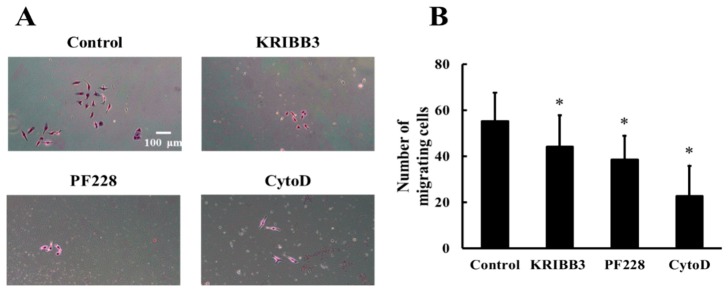
Effect of HSP27 on shear-stress-induced cell migration. (**A**) Shear-stress-induced cell migration images in the control, KRIBB3, PF228, and CytoD groups. (**B**) Comparison of cell migration numbers between the control and other groups under shear stress (* *p* < 0.05 when comparing to the control; *n* = 5 for all groups).

To investigate the effects of HSP27 phosphorylation, actin, and FAK activation on shear stress-induced cell migration, cells were pretreated with KRIBB3 (1 µM, 4 h), PF228 (50 nM, 30 min), or CytoD (1 µM, 30 min) prior to shear stress stimulations. The transwell migration assay was used to detect cell migration and the migrating cells without any inhibitor treatment were taken as the control. The results show that compared to the control group, the numbers of migrating cells in the KRIBB3, CytoD, and PF228 groups are significantly reduced (Figure 7A,B, *p* < 0.05), indicating that shear-stress-induced cell migration is affected by HSP27 phosphorylation, actin, and FAK activation.

## 4. Discussion

The HSP27 is over-expressed in a variety of cancer cells. Down-regulating HSP27 expression can reduce the area of tumor lesions and increase the sensitivity of tumor cells to chemotherapy and radiotherapy [18,44]. HSP27 expression and phosphorylation participate in the reorganization of the cytoskeleton with various drug stimulations, which may affect tumor cell motility [45], but the molecular mechanism of the effect of HSP27 on shear-stress-induced cell migration is not yet clear. In this study, it is found that shear stress induces HSP27 depolymerization into smaller active molecules from larger oligomers through its phosphorylation, which plays an essential role in HeLa cell polarity and migration. 

The spatial variation of HSP27 is related to the adaptation of cells to extracellular stimuli. It has been reported that HSP27 is mainly distributed in cytoplasm under normal conditions but it transfers into cell nuclei [46] to protect nucleic acid and improve cell viability under high heat stress stimuli [47]. In this study, the results show that HSP27 is uniformly distributed in cells under low shear stress stimuli while it accumulates downstream under higher shear stress stimuli. When cells migrate along the direction of flow, the cytoskeleton forms a new lamellipodia at the front of the cell which depends on the aggregation of actin and its related proteins at the cell front area [39,48,49]. It is speculated from the current finding that high shear stress may promote cell migration by enhancing the accumulation of HSP27 in the fluid direction and regulate the cytoskeletal rearrangement. Moreover, HSP27 and actin are colonialized in the lamellipodia of migrating cells [49], suggesting HSP27 involvement in the reorganization of the cytoskeleton at the front of the cell. Compared with high shear stress, cell alignment and migration are less affected by low shear stress, and the cytoskeletal changes gently. Consequently, HSP27 is uniformly distributed with low shear stress [45,50]. In addition, our results show that shear-stress-induced HSP27 polarity distribution is not related to its phosphorylation. This may be due to cytoskeletal reorganization at the front area of the cell that requires a large amount of actin monomer, whereas non-phosphorylated HSP27 can sequester the actin monomer [51]. Therefore, the non-phosphorylated HSP27 also clusters downstream in the cell. Noni T. Frankenberg et al. have also demonstrated that increases of HSP27 content in the cytoskeleton layer are independent of its own phosphorylation with extracellular stimuli [52].

As a capping protein of actin, the molecular chaperone function of HSP27 is regulated by its own polymerization [49,53]. The HSP27 oligomer can block the reorganization of the actin cytoskeleton, hinder the formation of lamellipodia and other structures at the cell front edge, and further influence cell migration [51,54]. HSP27 is a unique protein in the small heat shock protein family because it forms covalent disulfide bonds in homodimers [22]. These dimers are more easily formed with HSP27 phosphorylation. The three phosphorylation sites of HSP27 Ser15, Ser78, and Ser82 are located at the non-structural amino terminus of HSP27. These sites accumulate in the tertiary structure of the protein and are more exposed to the solvent [34]. Finally, through the interaction of negatively charged ions between the covalent bonds of the inner dimer, these sites can prevent HSP27 oligomerization [34]. Therefore, blocking HSP27 phosphorylation at different sites can inhibit shear-stress-induced depolymerization of HSP27. Our experimental results show that shear stress can reduce the degree of intracellular HSP27 polymerization, which depends on its own phosphorylation. Hence, it is suggested that shear stress can induce depolymerization of HSP27 through its own phosphorylation, inhibit the molecular chaperone function of HSP27 [54], and promote cytoskeletal reorganization.

Lamellipodia needs to be highly spatiotemporally coordinated with focal FAK in order to advance the cell front effectively during the cell migration process. Furthermore, the protruding edge is induced by new adhesion, which requires the participation of FAK, p130Cas, paxillin, and other proteins [55]. FAK promotes the polymerization of actin in primary focal adhesion by interacting with Arp2/3 and modulating the Rho/Rac activation cycle [56]. In addition, it promotes the formation of lamellipodia and filopodia in the cell front. The process requires a large number of actin monomers involved in the formation of these structures [48]. It is speculated that FAK polar activation and actin polar distribution induced by shear stress may stabilize the cell front. In addition, it has been found that the polarity of FAK is regulated by the phosphorylation of HSP27. Similarly, it has also been demonstrated that overexpression of phosphorylated variants of HSP27 promotes phosphorylation of FAK [57]. The HSP27 can increase cell adhesion, migration, and invasion ability through ß1 integrin expression and FAK phosphorylation [58], and reducing the phosphorylation of HSP27 may hinder the formation of lamellipodia [59]. It is clear that the inhibition of phosphorylation of HSP27 by drugs results in the decrease of FAK activation, after which interaction of FAK with Arp2/3 and the activation of the Rho/Rac cycle are blocked [60]. Afterward polarity of related molecules disappears, and finally, actin is uniformly distributed upon shear stress. It has been found in the above results that shear stress-induced reduction of HSP27 polymerization is dependent on FAK and actin regulation. HSP27 phosphorylation is regulated by MK5, which is a FAK substrate [61]. Shear-stress-induced FAK activation is related to cytoskeletons [29]. Thus, the shear stress-induced phosphorylation and depolymerization of HSP27 are also regulated by actin polymerization and FAK activation. All the above data show that the regulation of HSP27 in FAK and actin are not unidirectional, but mutual, and may play important roles in maintaining and stabilizing HSP27 polarity. 

## 5. Conclusions

Shear stress promotes the accumulation of HSP27 and actin in the downstream of cells in order to regulate cytoskeleton dynamics at the cell leading edge. In addition, shear stress also induces HSP27 depolymerization into small molecules by its phosphorylation, releasing new actin monomers, and then promoting the formation of new lamellipodia. Shear-stress-induced phosphorylation of HSP27 can promote FAK activation and facilitate the coordination of cytoskeleton reorganization and focal adhesion spatiotemporally, which affects the migration and adhesion of tumor cells. This research provides a molecular mechanism understanding for tumor cell adaption to external mechanical changes and suggests that HSP27 plays an important role in regulating shear-stress-induced tumor cell migration.

## Supplementary Materials

The following are available online at http://www.mdpi.com/2218-273X/9/2/50/s1.
