# Supplementary figures and images for: Heat Shock Protein 27 Phosphorylation Regulates Tumor Cell Migration under Shear Stress
Source: Biomolecules. 2019 Jan 30;9(2):50. doi: 10.3390/biom9020050 (PMC6406706; doi:10.3390/biom9020050)

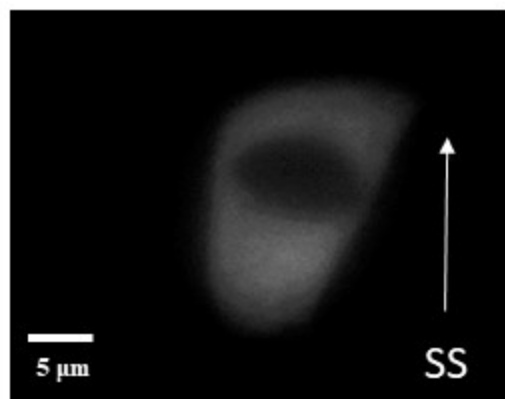

A

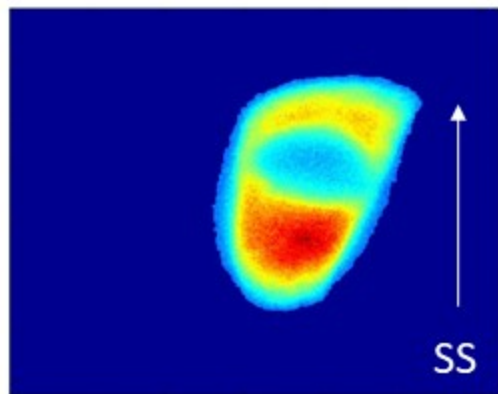

B

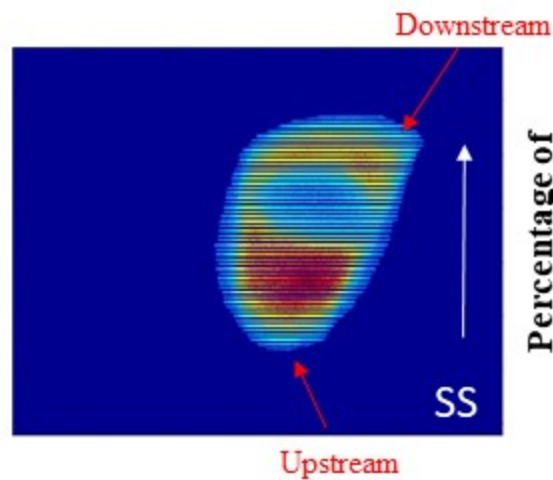

C

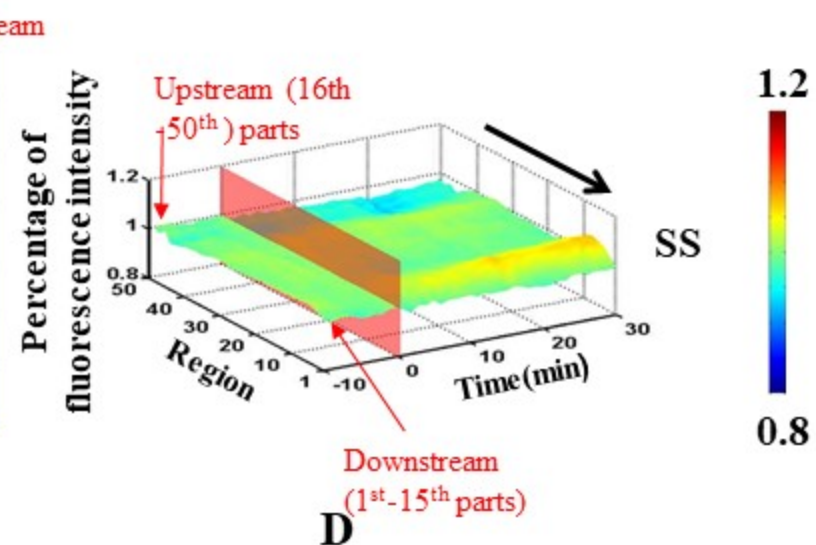

D

Supplement: Supplementary file 1 [file biomolecules-09-00050-s001.pdf]
